# Construction of a nomogram model for predicting peritoneal metastasis in gastric cancer: focused on cardiophrenic angle lymph node features

**DOI:** 10.1007/s00261-023-03848-7

**Published:** 2023-02-18

**Authors:** Xiaolong Gu, Yang Li, Gaofeng Shi, Li Yang, Hui Feng, Yang Yang, Zhidong Zhang

**Affiliations:** 1 Department of Radiology, The Fourth Hospital of Hebei Medical University, Shijiazhuang, 050000 Hebei PR China; 2 Department of Reproductive Medicine, The Second Hospital of Hebei Medical University, Shijiazhuang, 050000 China; 3 The Third Department of Surgery, The Fourth Hospital of Hebei Medical University, Shijiazhuang, 050000 China

**Keywords:** Cardiophrenic angle, Lymph node, Gastric cancer, Peritoneal metastasis, Prediction nomogram

## Abstract

**Background:**

A different treatment was used when peritoneal metastases (PM) occurred in patients with gastric cancer (GC). Certain cancers' peritoneal metastasis could be predicted by the cardiophrenic angle lymph node (CALN). This study aimed to establish a predictive model for PM of gastric cancer based on the CALN.

**Methods:**

Our center retrospectively analyzed all GC patients between January 2017 and October 2019. Pre-surgery computed tomography (CT) scans were performed on all patients. The clinicopathological and CALN features were recorded. PM risk factors were identified via univariate and multivariate logistic regression analyses. The receiver operator characteristic (ROC) curves were generated using these CALN values. Using the calibration plot, the model fit was assessed. A decision curve analysis (DCA) was conducted to assess the clinical utility.

**Results:**

126 of 483 (26.1%) patients were confirmed as having peritoneal metastasis. These relevant factors were associated with PM: age, sex, T stage, N stage, enlarged retroperitoneal lymph nodes (ERLN), CALN, the long diameter of the largest CALN (LD of LCALN), the short diameter of the largest CALN (SD of LCALN), and the number of CALNs (N of CALNs). The multivariate analysis illustrated that the LD of LCALN (OR = 2.752, *p <* 0.001) was PM’s independent risk factor in GC patients. The area under the curve (AUC) of the model was 0.907 (95% CI 0.872–0.941), demonstrating good performance in the predictive value of PM. There is excellent calibration evident from the calibration plot, which is close to the diagonal. The DCA was presented for the nomogram.

**Conclusion:**

CALN could predict gastric cancer peritoneal metastasis. The model in this study provided a powerful predictive tool for determining PM in GC patients and helping clinicians allocate treatment.

**Supplementary Information:**

The online version contains supplementary material available at 10.1007/s00261-023-03848-7.

## Introduction

On a global scale, gastric cancer (GC) ranked second in terms of mortality [1]. There was a 53% to 66% prevalence of peritoneal metastasis (PM) among patients with distant metastatic GC [2]. The quality of life of patients with PM was not satisfactory. Gastric cancer patients with PM should first receive neoadjuvant chemotherapy instead of direct surgical resection. At present, intraperitoneal hyperthermic perfusion chemotherapy had also been proven to help improve the prognosis. Immunotherapy and targeted therapy were also the frontier directions of current research. Accurate PM prediction was crucial. According to the direct signs of peritoneal metastasis, a new scoring system was developed to evaluate gastric cancer peritoneal metastasis [3]. Previous studies had constructed nomograms that include the collagen signature and tumor clinicopathological features to predict peritoneal metastasis [4]. Some studies had developed partial imaging models to predict peritoneal metastasis [5–8]. Deep learning models or machine learning models were also used to predict PM [9, 10]. In terms of different means of image examination, there was limited evidence about the effectiveness of magnetic resonance imaging (MRI) for gastric cancer staging [11–13]. On the other hand, positron emission tomography/ computed tomography (PET/CT) focused on the prediction of peritoneal metastasis based on new imaging agents or PET/CT imaging [14, 15]. However, the PET/CT examination was expensive and radioactive. In recent years, it had become not only necessary to predict the state of primitive cancer but also to pay attention to the risk factors of peritoneal metastasis after cure surgery [16].

Computed tomography (CT) was a regular noninvasive method of tumor node metastasis (TNM) staging. The typical signs of a diagnosis of PM were thickening of peritoneal cakes and massive ascites after excluding other diseases. These signs have high specificity but low sensitivity. However, the cases of gastric cancer with peritoneal metastasis without typical signs were confirmed to have small metastatic nodules after diagnostic laparoscopic exploration and could exist in isolation. Therefore, other methods were needed to judge the status of PM. The cardiophrenic angle lymph node (CALN) was effective in predicting rectal and ovarian cancer peritoneal metastasis [17, 18]. Especially for ovarian cancer, the cardiophrenic angle lymph nodes showed predictive value in diagnosis and prognosis [19–21]. Moreover, CT scanning of cardiophrenic lymph nodes could be performed at the same time as routine abdominal CT scanning, without the need to book additional examinations again. There was a lack of a PM model for GC patients that focused on the CALN [22].

Accurate preoperative prediction of PM had clinical significance for the accurate selection of a treatment plan. This study aimed to establish a predictive model of gastric cancer peritoneal metastasis focusing on cardiophrenic lymph nodes.

## Methods

### Patient population and study design

**Fig. 1:**
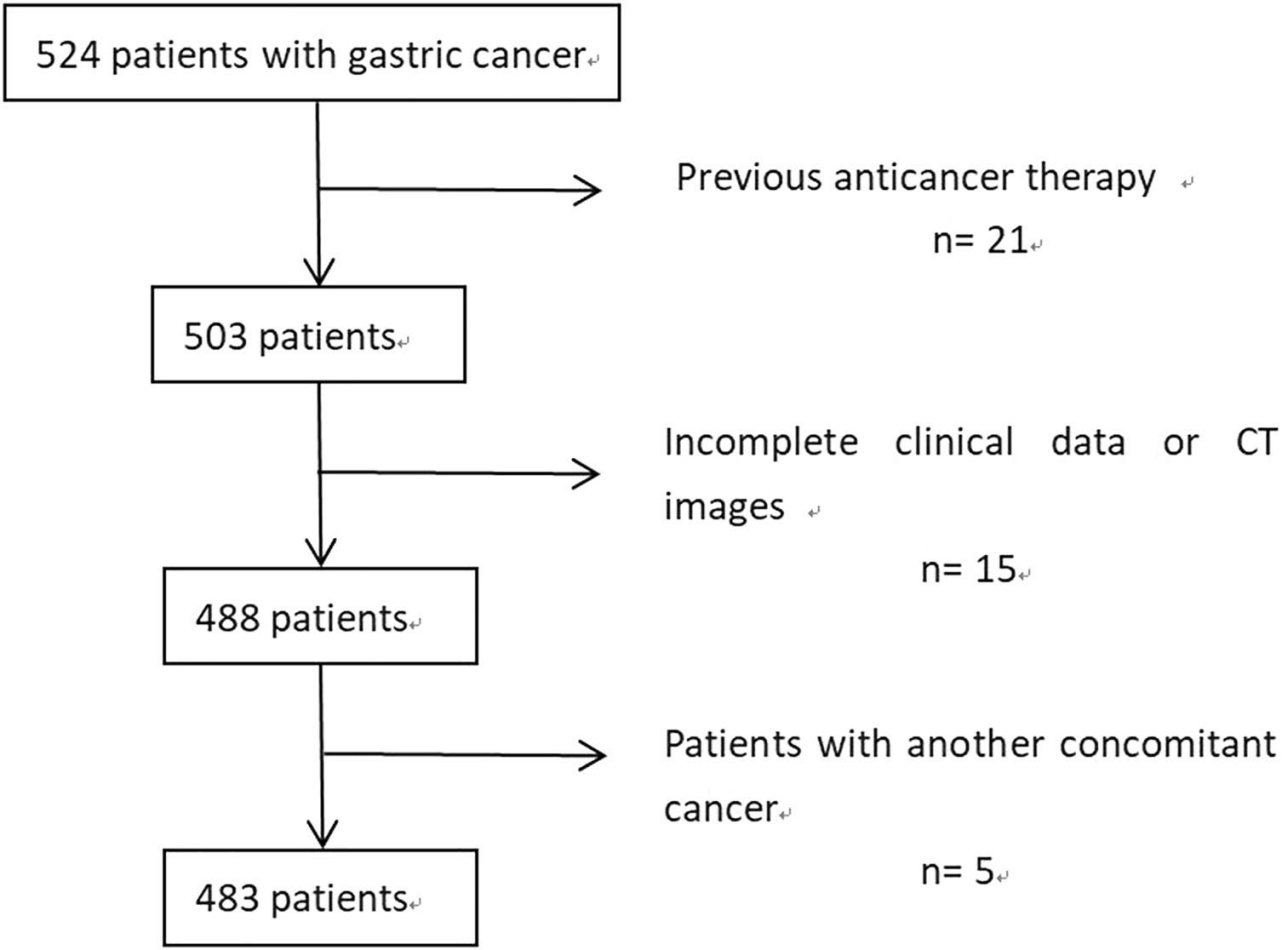
Flowchart of the study

The ethics committee approved this retrospective study (protocol number 2021KY120). As this was a retrospective study using deidentified data, written informed consent was waived. Our center retrospectively analyzed all GC patients between January 2017 and October 2019. The inclusion criteria were: [1] GC confirmed by pathology; [2] within two weeks before surgery, a CT scan was performed; [3] the status of PM was confirmed by surgery. The exclusion criteria were (Fig. 1): [1] prior anticancer therapy; [2] insufficient clinical data or CT images; and [3] patients with another cancer.

### The clinical evaluation index

The clinical characteristics were recorded: age, sex, location, liver metastasis (LM), enlarged retroperitoneal lymph nodes (ERLN), enlarged lymph node in the left supraclavicular fossa (ELSFLD), T stage, N stage, and peritoneum metastasis (PM). Clinical staging was used in the T stage and the N stage. For ERLN and ELSFLD, we define that the short diameter was greater than 10 mm. The diameters were measured on the horizontal section CT images.

### CT acquisition technique

All CT examinations were performed using one of three multidetector CT scanners: a 256-detector CT scanner (Revolution CT, GE Medical Systems) and two 128-detector CT scanners (SOMATOM Definition Flash, Siemens Healthcare, and Brilliance iCT, Philips Healthcare). The scans included unenhanced chest scans and multiphase contrast-enhanced abdominal scans to cover the cardiophrenic angle. The arterial phase and venous phase were scanned at 25 s and 70 s after injection. An automatic milliampere second technology was used to measure tube current while voltage was 120 kV. The reconstruction thickness was 1.0 mm because the measurement target is at the millimeter level.

### Image analysis

Two radiologists (GXL has 7 years of experience in abdominal radiology, while YL has 19 years) retrospectively analyzed the CALN independently. Disagreements were resolved through consensus. CT features on the entire cohort were recorded: [1] the presence of CALN, the long diameter of the largest CALN (LD of LCALN), the short diameter of the largest CALN (SD of LCALN), and the number of CALNs (N of CALNs); and [2] the presence of peritoneal carcinoma signs. PM status was blinded to the radiologist.

### Statistical analysis

Chi-square or Mann–Whitney U tests were used to compare groups. *P <* 0.05 indicated statistical significance. PM risk factors were identified via univariate and multivariate logistic regression analyses. The receiver operator characteristic (ROC) curves were generated using these CALN values. Using the calibration plot, the model fit was assessed. A decision curve analysis (DCA) was conducted to assess the clinical utility. All statistical analyses were performed using R version 3.6.3 and Python version 3.7.

## Results

### Patient characteristics

For peritoneal metastasis, 126 of 483 (26.1%) patients were positive while 357 patients were negative (Table 1). Study participants had an average age of 61 years.

**Table 1:** Demographic and clinical characteristics of the study population

		All	Negative	Positive	*p*-value
		(*N =* 483)	(*N =* 357)	(*N =* 126)	
Sex, N(%)	Male	347(71.8)	267(74.8)	80(63.5)	0.015
	Female	136(28.2)	90(25.2)	46(36.5)	
Location, N(%)	Cardia	68(14.1)	53(14.8)	15(11.9)	0.157
	Fundus	14(2.9)	13(3.6)	1(0.8)	
	Body	162(33.5)	112(31.4)	50(39.7)	
	Antrum	239(49.4)	179(50.1)	60(47.6)	
T stage, N(%)	T1–3	63(13.0)	55(15.4)	8(6.3)	0.009
	T4	420(87.0)	302(84.6)	118(93.7)	
N stage, N(%)	N0	97(20.1)	83(23.2)	14(11.1)	0.005
	N1	132(27.3)	100(28.0)	32(25.4)	
	N2	154(31.9)	110(30.8)	44(34.9)	
	N3	100(20.7)	64(17.9)	36(28.6)	
LM, N(%)	Negative	456(94.4)	337(94.4)	119(94.4)	0.984
	Positive	27(5.6)	20(5.6)	7(5.6)	
ERLN, N(%)	Negative	435(90.1)	333(93.3)	102(81.0)	< 0.001
	Positive	48(9.9)	24(6.7)	24(19.0)	
ELSFLD, N(%)	Negative	477(98.8)	353(98.9)	124(98.4)	0.684
	Positive	6(1.2)	4(1.1)	2(1.6)	
CALN, N(%)	Negative	242(50.1)	228(63.9)	14(11.1)	< 0.001
	Positive	241(49.9)	129(36.1)	112(88.9)	
SD of LCALN,median[IQR]	–	0.0[0.0,3.0]	0.0[0.0,2.0]	3.0[2.0,4.0]	< 0.001
LD of LCALN,median[IQR]	–	0.0[0.0,5.0]	0.0[0.0,4.0]	6.0[4.0,7.0]	< 0.001
N of CALNs,median[IQR]	–	0.0[0.0,1.0]	0.0[0.0,1.0]	1.0[1.0,2.0]	< 0.001
Age, median[IQR]	–	61.0[54.0,67.0]	61.0[55.0,68.0]	60.0[54.0,66.0]	0.031

*LM* liver metastasis, *ERLN*, enlarged retroperitoneal lymph nodes, *ELSFLD* enlarged lymph node in the left supraclavicular fossa, *CALN* cardiophrenic angle lymph node, *SD* of *LCALN *the short diameter of the largest CALN, *LD* of *LCALN* the long diameter of the largest CALN, *N* of *CALNs* the number of CALNs, *IQR* interquartile range

### Univariate and multivariate analysis for PM

For CALN, 241 of 483 (49.9%) patients were positive while 242 patients were negative. For ERLN, 48 of 483 (9.9%) patients were positive while 435 patients were negative. For ELSFLD, 6 of 483 (1.2%) patients were positive while 477 patients were negative. According to univariate logistic regression analysis (Table 1 and Table 2), the following relevant factors were associated with PM (*p <* 0.05): age, sex, T stage, N stage, ERLN, CALN, SD of LCALN, LD of LCALN, and N of CALNs. The multivariate analysis illustrated that the LD of LCALN (OR = 2.752, *p <* 0.001) and sex (OR = 2.663, *p =* 0.004) were PM’s independent risk factors in GC patients (Supplementary Table 2).

**Table 2:** Logistic regression analysis of the risk factors for peritoneal metastasis

	Number	OR	95%CI	p-value
N of CALNs	483	4.89	[3.49,6.85]	0
LD of LCALN	483	1.92	[1.69,2.17]	0
SD of LCALN	483	2.48	[2.08,2.95]	0
Age				
≤ 60y	204			
> 60y	279	0.81	[0.54,1.22]	0.316
Sex				
Male	347			
Female	136	1.71	[1.11,2.63]	0.016
Location				
Cardia	68			
Fundus	14	0.27	[0.03,2.25]	0.227
Body	162	1.58	[0.81,3.06]	0.178
Antrum	239	1.18	[0.62,2.25]	0.606
T stage				
T1-3	63			
T4	420	2.69	[1.24,5.81]	0.012
N stage				
N0	97			
N1	132	1.90	[0.95,3.79]	0.07
N2	154	2.37	[1.22,4.61]	0.011
N3	100	3.34	[1.66,6.70]	0.001
LM				
Negative	456			
Positive	27	0.99	[0.41,2.40]	0.984
ERLN				
Negative	435			
Positive	48	3.27	[1.78,6.00]	0
ELSFLD				
Negative	477			
Positive	6	1.42	[0.26,7.87]	0.686
CALN				
Negative	242			
Positive	241	14.14	[7.79,25.66]	0

*LM* liver metastasis, *ERLN* enlarged retroperitoneal lymph nodes, *ELSFLD* enlarged lymph node in the left supraclavicular fossa, *CALN* cardiophrenic angle lymph node, *SD* of *LCALN* the short diameter of the largest CALN, *LD* of *LCALN* the long diameter of the largest CALN, *N* of *CALNs* the number of CALNs, *OR* odds ratio, *CI* confidence interval

### ROC curve of risk factors

**Fig. 2:**
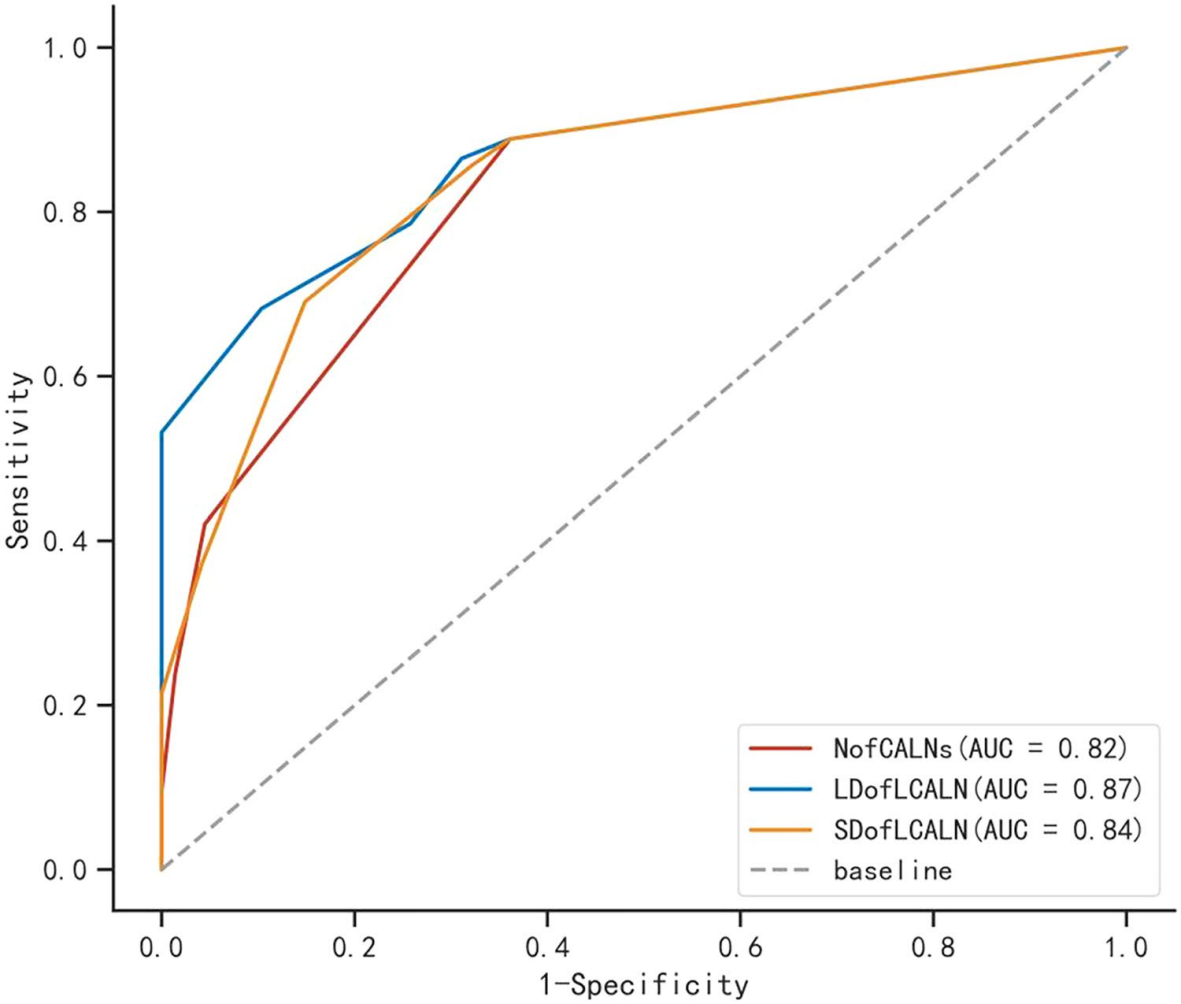
Receiver operating curve (ROC) curves of the risk factors. *CALN* cardiophrenic angle lymph node, *NofCALNs* the number of CALNs, *LDofLCALN* the long diameter of the largest CALN, *SDofLCALN* the short diameter of the largest CALN

A correlation was determined between PM risk and CALN features (N of CALNs, LD of LCALNs, and SD of LCALNs) in GC patients using ROC curves (Fig. 2). The area under the curve (AUC), specificity, and sensitivity values were presented in Supplementary Table 1.

### Multivariate predictive model and nomogram for predicting PM of GC

**Fig. 3:**
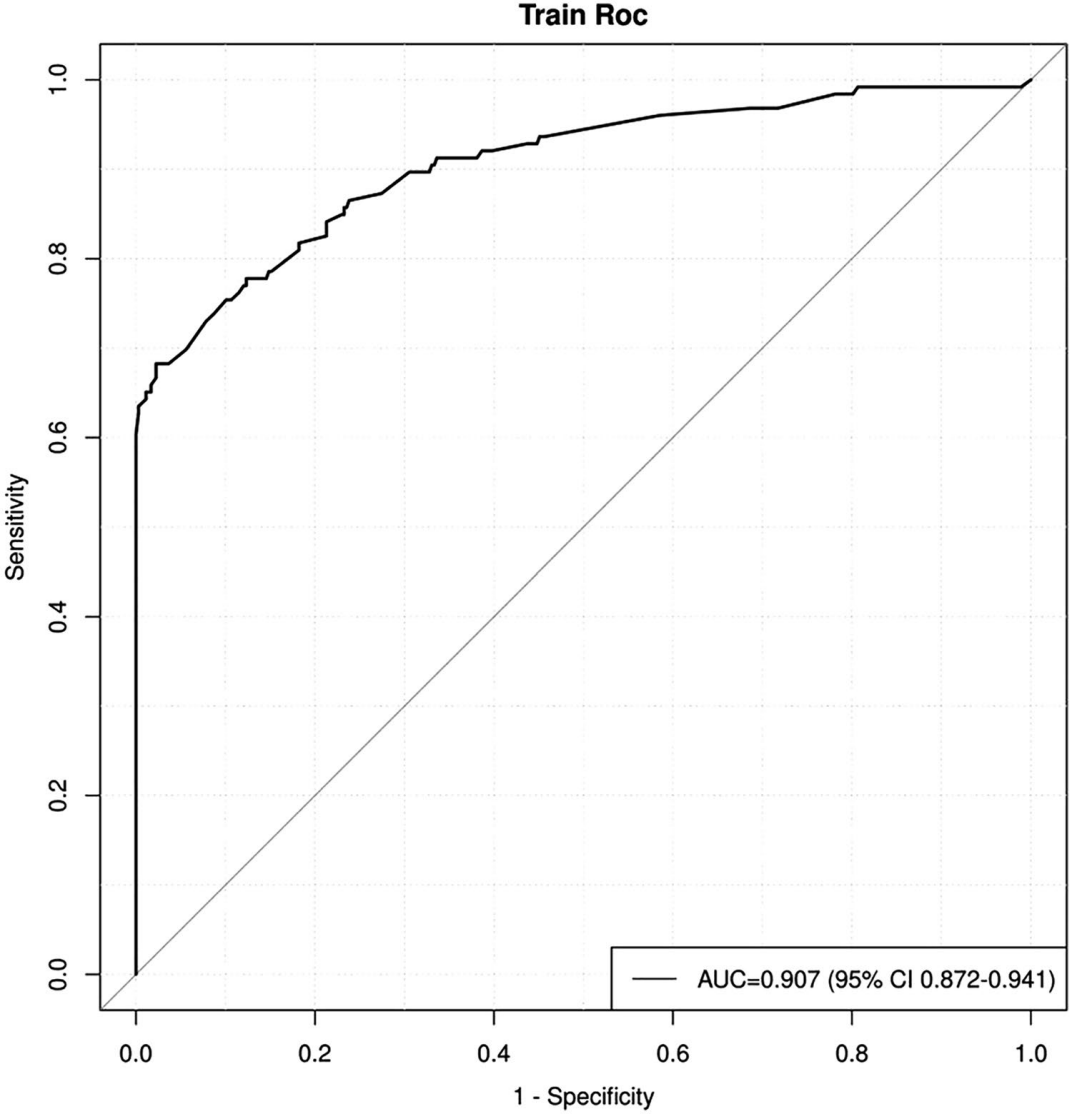
Receiver operating curve (ROC) curves of the multivariate logistic regression model. NofCALNs, number of CALNs; AUC, area under the curve; CI, confidence interval

The AUC of the model was 0.907 (95% CI: 0.872–0.941), demonstrating good performance in the predictive value of PM (Fig. 3) (Supplementary Table 2).

**Fig. 4:**
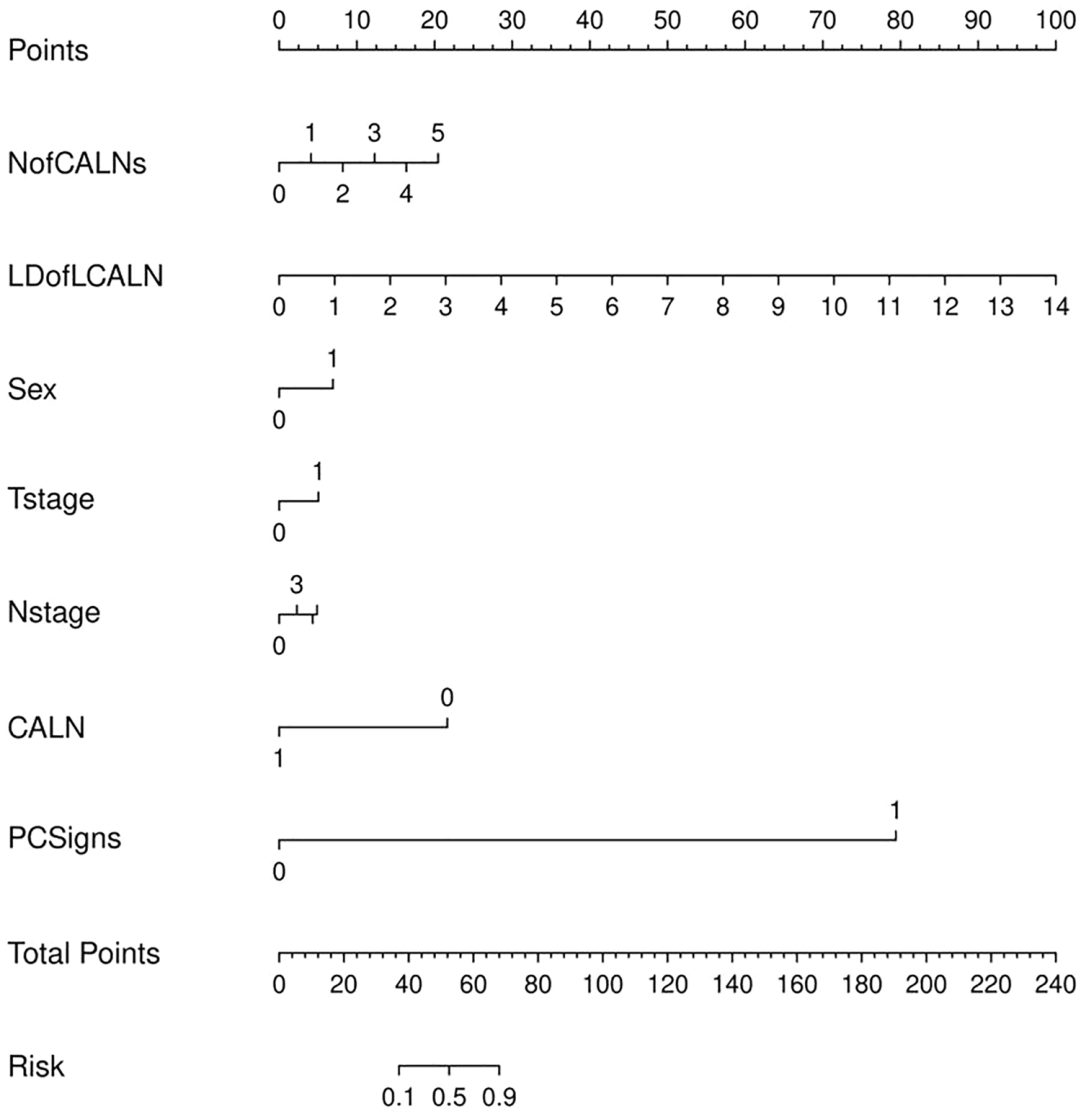
Gastric cancer patients’ PM probability could be predicted using a nomogram. *CALN* cardiophrenic angle lymph node, *LDofLCALN* long diameter of the largest CALN, *NofCALNs* number of CALNs, *PCSigns* peritoneal carcinoma signs

**Fig. 5:**
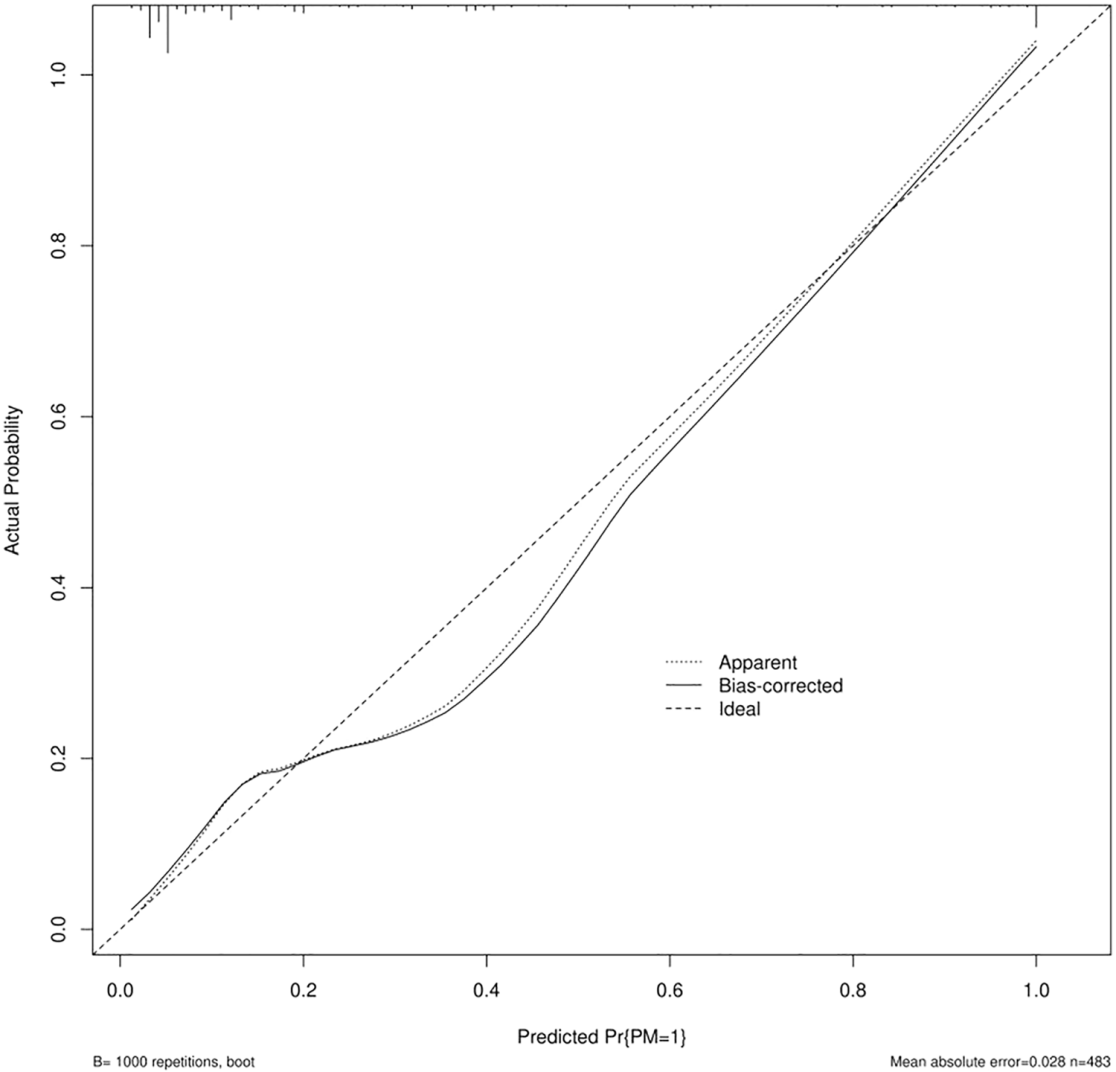
The calibration plot of the nomogram model. The solid line is the bias-corrected line. The dashed lines represent the ideal line and the apparent line. There is excellent calibration evident from the calibration plot, which is close to the diagonal

**Fig. 6:**
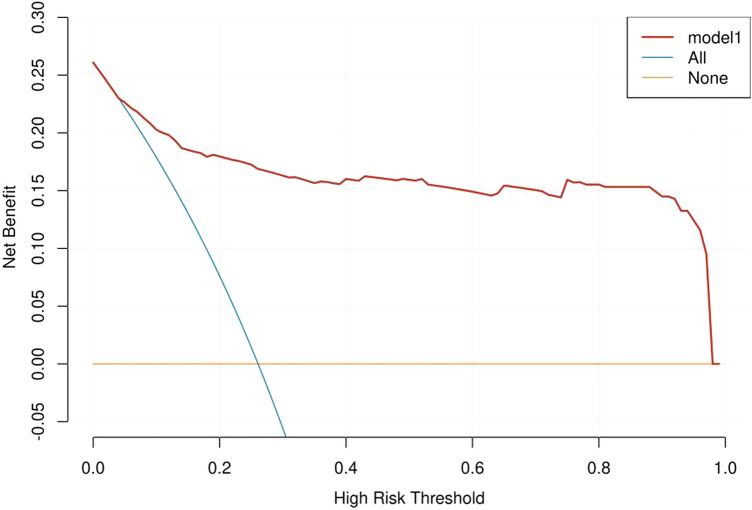
DCA curve of the model. The model prediction effect is shown by the area under the curve

The nomogram of PM incidence was constructed (Fig. 4). There is excellent calibration evident from the calibration plot (Fig. 5), which is close to the diagonal. The DCA (Fig. 6) was presented to indicate that the model was clinically beneficial.

## Discussion

CT was a regular method to diagnose PM of GC. The value of CALN in the prediction of PM has been continuously revealed. Our research found that combining CT with clinical factors could be used to create a predictive model.

We studied the value of clinicopathological features and CALN features as markers of GC with PM. Multivariate logical regression analysis showed that the long diameter of the largest CALN was an independent risk factor. Nomograph provided visual data information. A nomogram based on clinicopathological factors (sex, T stage, N stage) and CT signs of CALN (N of CALNs, LD of LCALN, CALN) could predict PM. There is excellent calibration evident from the calibration plot. The model was clinically beneficial by DCA. The nomogram in this study could accurately predict PM status in CG patients and helped make a more accurate treatment plan for GC patients.

The lymph node of the cardiophrenic angle was an important basis for clinical diagnosis and prognosis evaluation of tumors. Previous studies have shown that the cardiophrenic angle lymph node is an important predictor in the diagnosis and progression monitoring of digestive tract and ovarian malignant tumors [17–19]. Compared with the previous model, this study innovatively focused on the CT characteristics of CALN [22]. The diagnostic effectiveness of this study was similar to or even better than some imaging or machine learning models [5, 7, 23, 24]. The characteristics of CPLN were also analyzed in this study. Through the addition of quantitative indicators, the prediction effect of the model had been greatly improved. Other studies on the CALN in ovarian cancer had also confirmed the significance of the size of the CALN in the diagnosis of PM [20, 21]. This revealed the significance of quantitative indicators in the evaluation of CALN.

As far as the scanning technique was concerned, the examination of CALN was simple and easy. The guidelines recommended a chest scan to check for lung metastasis [25]. The examination of the cardiophrenic angle could be completed at the same time. However, CT scanning of CALN did not need the injection of a contrast agent. And the CT image of CALN was displayed because of the natural background, so it was easy to measure. The boundary of the cardiophrenic angle lymph node was clear, the shape was regular, and the measurement result was more reliable.

Several related research directions were pursued. There had been a gradual understanding of the molecular mechanism underlying gastric cancer peritoneal metastasis. A non-negligible role was played by lipid metabolism in epithelial-mesenchymal transition (EMT), which was crucial to gastric cancer metastasis [26]. A cancer cell's ability to metastasize was affected by the presence of collagen in its microenvironment [4]. The cardiophrenic lymph node was the relay station of lymphatic drainage, and the cardiophrenic lymph node could be enlarged due to tumors in patients with peritoneal metastasis. The relationship between the molecular mechanism of GC peritoneal metastasis and CALN enlargement was worthy of further study. In addition, it was expected that the CALNs could be pathologically verified by cardiophrenic angle lymphadenectomy [27].

In our research, there were some limitations. Firstly, as a retrospective study conducted in a single subspecies center, it was not certain that it applies to all ethnic groups. Secondly, a slight increase in peritoneal fat density was not used as a feature of peritoneal metastasis because it might be suggestive but lacks specificity.

In conclusion, CALN could predict gastric cancer peritoneal metastasis. The model in this study provided a powerful predictive tool for determining PM in GC patients and helping clinicians allocate treatment.

## Supplementary Information

Below is the link to the electronic supplementary material.

Supplementary file1 (DOCX 18 KB)
