# Supplementary material for: Construction of a nomogram model for predicting peritoneal metastasis in gastric cancer: focused on cardiophrenic angle lymph node features
Source: Abdom Radiol (NY). 2023 Feb 18;48(4):1227–36. doi: 10.1007/s00261-023-03848-7 (PMC10115726; doi:10.1007/s00261-023-03848-7)
Supplement: Supplementary file 1 — Supplementary file1 (DOCX 18 KB) [file 261_2023_3848_MOESM1_ESM.docx]

Construction of a Nomogram Model for Predicting Peritoneal Metastasis in Gastric Cancer: Focused on Cardiophrenic Angle Lymph Node Features

Supplementary Table 1  The AUC, specificity, and sensitivity values of the receiver operating curve (ROC) curves of cardiophrenic angle lymph node features

| Characteristics | AUC | Sensitivity | Specificity | Youden Index | Optimal threshold |
| --- | --- | --- | --- | --- | --- |
| N of CALNs | 0.823 | 0.889 | 0.639 | 0.528 | 1 |
| LD of LCALN | 0.867 | 0.683 | 0.896 | 0.579 | 5 |
| SD of LCALN | 0.841 | 0.690 | 0.852 | 0.542 | 3 |

Abbreviations: SD of LCALN, short diameter of the largest CALN; LD of LCALN, long diameter of the largest CALN; N of CALNs, number of CALNs; AUC, area under the curve.

Supplementary Table 2 Multivariate analysis results

| Predictor | Estimate | SE | Z | p | Odds Ratio | Lower | Upper |
| --- | --- | --- | --- | --- | --- | --- | --- |
| (Intercept) | -4.372 | 0.695 | -6.295 | 0.000 | 0.013 | 0.003 | 0.045 |
| N of CALNs | 0.580 | 0.307 | 1.890 | 0.059 | 1.786 | 0.990 | 3.335 |
| LD of LCALN | 1.012 | 0.174 | 5.805 | 0.000 | 2.752 | 2.009 | 3.998 |
| Female | 0.980 | 0.342 | 2.867 | 0.004 | 2.663 | 1.363 | 5.235 |
| T4 | 0.714 | 0.557 | 1.283 | 0.199 | 2.043 | 0.744 | 6.805 |
| N1 | 0.610 | 0.491 | 1.242 | 0.214 | 1.840 | 0.721 | 5.019 |
| N2 | 0.691 | 0.479 | 1.441 | 0.149 | 1.996 | 0.801 | 5.333 |
| N3 | 0.322 | 0.557 | 0.579 | 0.563 | 1.380 | 0.463 | 4.195 |
| CALN Positive | -3.067 | 0.928 | -3.306 | 0.001 | 0.047 | 0.007 | 0.255 |

Abbreviations: CALN, cardiophrenic angle lymph node; LD of LCALN, long diameter of the largest CALN; N of CALNs, number of CALNs.
